# Species-specific difference of CO₂ emissions in mangroves: coupling sediment physicochemistry and microbial communities

**DOI:** 10.3389/fmicb.2025.1694593

**Published:** 2025-10-29

**Authors:** Ziying He, Xueyin Zhuang, Jin Liang, Yisheng Peng, Huaye Sun, Zhushi Yin, Meng Xia, Lili Zhao, Bowen Hu, Ming Qu, Weidong Zhu

**Affiliations:** ^1^Guangdong Forestry Survey and Planning Institute, Guangzhou, China; ^2^School of Environmental Science and Engineering, Sun Yat-Sen University, Guangzhou, China; ^3^Institute of Carbon Neutrality and Green Development, Sun Yat-sen University, Guangzhou, China; ^4^Guangdong Marine Development Planning Research Center, Guangzhou, China; ^5^Zhuhai Western Ecological Environment Monitoring Center, Zhuhai, China

**Keywords:** mangrove, *Kandelia obovate*, *Sonneratia apetala*, sediment CO_2_ fluxes, root-respiration CO_2_ fluxes, microbial communities

## Abstract

Mangrove ecosystems function simultaneously as carbon sinks and carbon sources. While their contribution to biomass accumulation and long-term carbon sequestration have been extensively studied, the mechanisms driving carbon emissions, particularly those mediated by tree species and microbial communities, remain poorly understood. In this study, we investigated *Kandelia obovate* (KO), *Sonneratia apetala* (SA), and an adjacent mudflat in the Hanjiang River Estuary, southern China, to evaluate seasonal changes in sediment physicochemistry, microbial community structure, and CO₂ fluxes, and to evaluate the influence of vegetation on carbon emissions. This research shows that mangrove colonization significantly altered sediment conditions, with *K. obovata* exhibiting higher salinity, water content, and total carbon concentration than *S. apetala*. Sediment CO₂ fluxes were consistently greater in mangrove habitats than in mudflats and displayed clear seasonal variation. In summer, sediment CO₂ fluxes in *S. apetala* and *K. obovata* were 4.3- and 2.5-fold higher than in winter, respectively. Concurrently, root respiration intensified in *S. apetala* during summer, whereas *K. obovata* root respiration remained stable across seasons. Microbial communities were dominated by Proteobacteria and Chloroflexi across sites, however, their network structures differed. *S. apetala* supported tighter microbial interactions, while *K. obovata* exhibited higher modularity and functional specialization. Additionally, partial least squares structural equation modeling revealed that sediment physicochemical properties strongly constrained microbial diversity and regulated CO₂ flux both directly and indirectly. These findings highlight the importance of sediment and root respiration in mangrove carbon cycling and demonstrate how species identity modulates CO₂ fluxes by shaping the interactions between sediment conditions and microbial communities.

## 1 Introduction

Mangrove ecosystems play a pivotal role in the global carbon cycle, functioning as highly efficient carbon sinks due to their exceptional primary productivity and long-term carbon sequestration in both biomass and sediments (Alongi, 2014). Previous estimates suggest that mangroves store 3–5 times more carbon per unit area than terrestrial forests, with sediment organic carbon (SOC) pools persisting for millennia (Alongi, 2014; Macreadie et al., 2021). However, a substantial fraction of the carbon sequestered in mangroves is subsequently released to the atmosphere as CO_2_ through heterotrophic respiration and porewater exchange. Global assessments indicate that mangrove-derived CO_2_ fluxes average 125 ± 18 Tg CO_2_ year^−1^ (equivalent to 34 ± 5 Tg C year^−1^), slightly exceeding their estimated long-term carbon burial rate (26 Tg C year^−1^) (Reithmaier et al., 2020). This imbalance underscores the dual role of mangroves as both carbon sinks and sources.

The production and export of greenhouse gases from mangrove sediments exhibit pronounced spatial and temporal variability, regulated by a range of biotic and abiotic factors. Key determinants include species composition, stand age, tidal zone position, seasonal variations, tidal cycles, and the composition and abundance of sediment microbial communities (Kristensen et al., 2017; Leopold et al., 2013). Specifically, CO_2_ fluxes at the sediment–air interface is influenced by sediment characteristics, biogenic structures (such as crab burrows and pneumatophores), and microbial assemblages (Lovelock, 2008; Chen G. C. et al., 2012; Das et al., 2016; Gillis et al., 2017; Ouyang et al., 2017). Sediment-derived CO_2_ emissions represent a major carbon loss pathway, directly affecting sediment carbon storage capacity, and should therefore be considered in global carbon budget assessments.

In China, mangrove coverage has declined drastically: a national survey reported that by 2002, only 44% of the mangrove area present in the 1950s remained (Jia et al., 2018). Recognizing this loss, the Chinese government has implemented a series of restoration and conservation initiatives since the 1990s. *Sonneratia apetala* Buch.-Ham., a mangrove species originally introduced from Bangladesh to China in 1985, has since been widely employed in mangrove afforestation programs owing to its remarkable adaptability and rapid growth rate (He et al., 2018; Dong et al., 2025). Recent studies indicate that *S. apetala* is predominantly distributed in Guangdong province, covering a total area of 4000.4 ha, which represents approximately 14.8% of the total mangrove area in China as of 2022 (Zhao et al., 2024; Dong et al., 2025). *Kandelia obovata* Sheue, H. Y. Liu & J. Yon is a native mangrove species predominantly distributed along the southeastern coastline of China, with Guangdong province supporting the largest contiguous communities of this species (Chen L. et al., 2025; Chen J. et al., 2025). In the Hanjiang River Estuary, Guangdong province, China, seedlings of the exotic *S. apetala* and the native *K. obovata* were planted 3 m apart on intertidal flats composed of 4.7% sand, 88.2% silt, and 7.1% clay, within a similar tidal range (1.45–1.55 m). The average flooding durations were 10.3 h for *K. obovata* and 10.6 h for *S. apetala* (He et al., 2018). After 13 years, *K. obovata* and *S. apetala* plantations expanded to 3.27 ha and 9.39 ha, respectively, providing diverse ecosystem services, particularly as carbon sinks.

Our previous study revealed that mean SOC stocks in planted *K. obovata* stands reached approximately 15.8 Mg ha^−1^, compared with 7.8 Mg ha^−1^ in *S. apetala* plantations, while adjacent unvegetated mudflats contained only 4.7 Mg ha^−1^ (He et al., 2020). The high carbon burial rate in this region has been attributed either to a low export of net primary productivity or, more plausibly, to a high sediment accumulation rate. Such exceptionally high carbon burial may, in turn, contribute to substantial CO_2_ fluxes from mangroves to the atmosphere, particularly across the surface sediment.

Despite the ecological significance of this process, data on greenhouse gases, especially CO_2_ production and emissions from this area, remain scarce. To date, the only study addressing sediment-derived CH₄ fluxes and their controlling factors focusing on sediment properties and methanogenic and methanotrophic communities was conducted by Yu et al. (2020) in this region. The present study investigated CO_2_ fluxes from both sediments and mangrove fine roots, alongside measurements of sediment physicochemical properties and microbial community composition and abundance, to integrate the influence of biotic and abiotic factors governing carbon fluxes in mangrove ecosystems.

## 2 Materials and methods

### 2.1 Site description and sediment sampling

**Figure 1:**
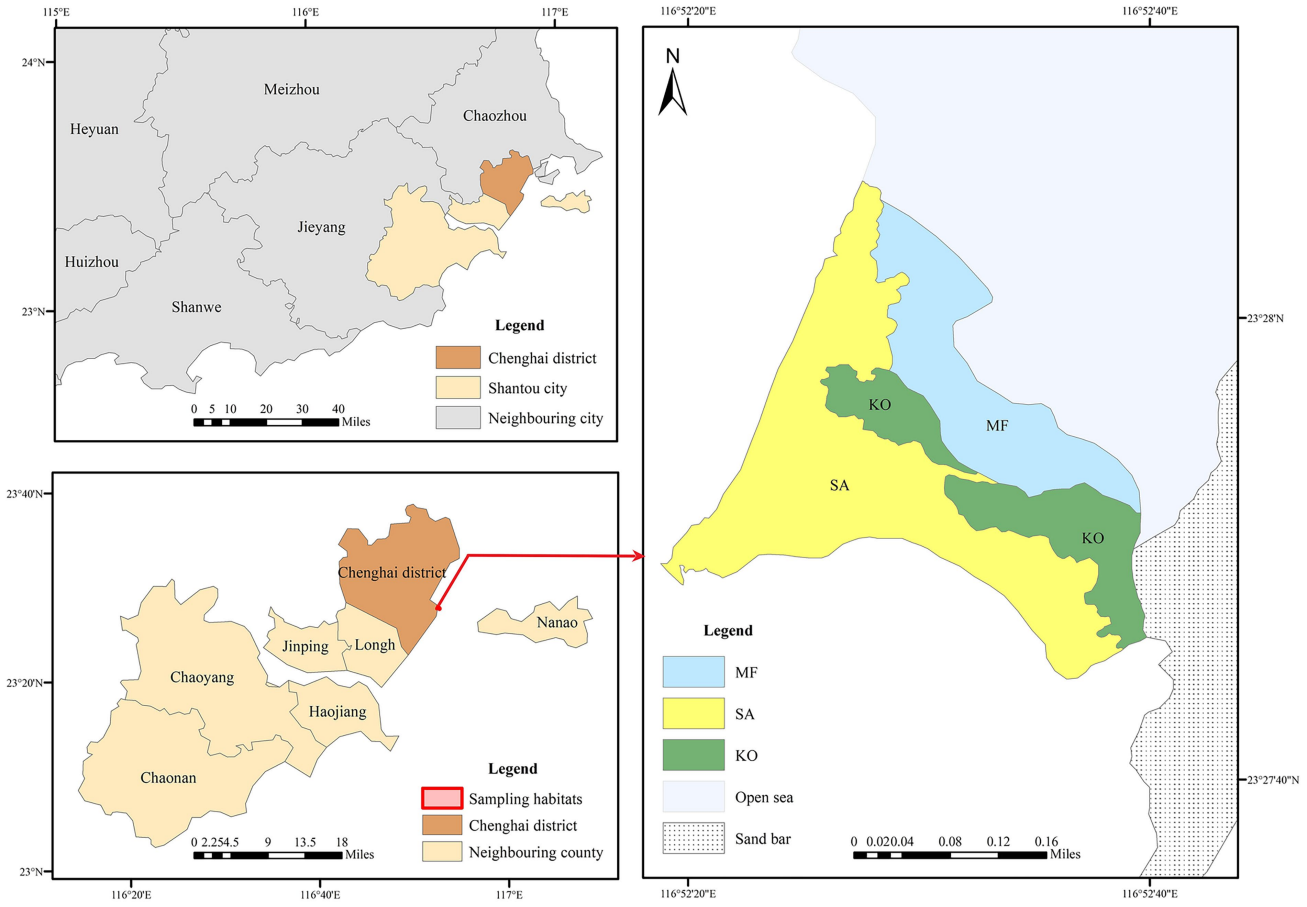
The location of sampling habitats at Hanjiang River Estuary, Guangdong Province, southern China.

The sampling site is situated in the Hanjiang River Estuary (23.45°N, 116.43°E), Guangdong Province, southern China (Figure 1). Sediment samples were collected from two distinct mangrove stands: a native *K. obovata* (KO) plantation and an introduced *S. apetala* (SA) plantation. Additionally, samples were obtained from an adjacent unvegetated mudflat, which served as a control site for comparative analysis. Sediment sampling was conducted in April 2018, during which six 10 m × 10 m plots were established in each habitat type. Sediment cores were collected using a custom-made sampler and subsequently sectioned into four vertical depth intervals: 0–5, 5–10, 10–15, and 15–20 cm, yielding a total of 72 samples. All samples were immediately stored in a portable cooler maintained at 4 °C and transported to the laboratory within 24 h of collection. Upon arrival, each sample was sliced into two equal portions: one portion was preserved at 4 °C for subsequent analysis of physicochemical properties, while the other portion was stored at −80 °C for future DNA extraction.

### 2.2 Gas emissions and root respiration

*In situ* measurements of sediment gas emission and root respiration were conducted during 2018–2019, covering April (spring), July (summer), October (autumn), and January (winter). Sediment gas emissions were measured during low tide period using six static chambers (20 cm × 20 cm × 20 cm) connecting to connected to a portable gas analyzer (AZG300, Aozuo instrument, China) for simultaneous quantification of CO_2_ fluxes. During deployment, the open end of each chamber was carefully inserted 1–2 cm into the sediment at each sampling location. Gas concentrations were monitored over a 30-min period, with measurements recorded at 10-min intervals.

The sediment CO_2_ fluxes (${\text{Flux}}_{{\mathrm{CO}}_{2}}$) were calculated as:


$${\text{Flux}}_{{\mathrm{CO}}_{2}}=\frac{K*\mathrm{ppm}}{\sec}$$

where ppm is the variation in the molar concentration of CO_2_ in the static chamber; sec is the gas passage time (600 s); the calculation formula for the gas constant K is as follows:


$$K=\frac{86,400*P}{10^{6}*R*T_{k}}*\frac{V}{A}$$

where P is the barometric pressure expressed in mBar (1.013 Hpa); R is the gas constant (0.083145 bar L K^−1^ mol^−1^); $T_{k}$ is the air temperature expressed in Kelvin degree (298 K); V is the chamber net volume in m^3^ (0.008 m^3^); A is the chamber inlet net area in m^2^ (0.04 m^2^).

Fine root respiration measurements were performed on roots (diameter ≤2 mm) collected from top the 50 cm of sediment near mangrove stems in the study area. Fine roots were selected for respiration measurement as they represent the most metabolically active fraction of the root system and are the primary contributors to belowground carbon fluxes (He et al., 2021). The roots were rinsed with seawater to remove adhering sediment. Root samples (between 0.05 and 0.4 g dry mass) were loosely wrapped in seawater-moistened tissue paper and immediately placed in an aluminum cuvette (5.4 cm diameter × 15 cm length) attached to the same gas analyzer (Lovelock et al., 2006). To maintain ambient temperature, the cuvette was submerged in seawater (Lovelock et al., 2006). Before measurements, roots were allowed to acclimate for 5 min until CO₂ exchange rates stabilized. Gas exchange was then recorded every 30 s for 7.5 min. The calculation formula for root-respiration CO_2_ fluxes is consistent with that for sediment CO_2_ emission fluxes. The respiration rate for each sample was calculated as the mean of 15 logged values, with three replicates per mangrove species. Additionally, control measurements (tissue paper moistened with seawater but without roots) were conducted to account for background gas exchange.

It is important to note that sediment sampling for physicochemical and microbial analysis was conducted only in April 2018. While gas fluxes were measured seasonally, this single time-point sampling for sediments was designed to capture the inherent, habitat-specific differences driven by long-term vegetation presence, which are relatively stable compared to more dynamic flux measurements.

### 2.3 Sediment physicochemical properties analysis and microbial community DNA extraction

In the laboratory, air-dried sediment samples were finely ground prior to physicochemical analyses. For pH determination, 2.0 g of dried sediment was mixed with deionized water at a 1:2.5 (w/v) ratio and measured using a pH meter (SevenCompact210, Mettler-Toledo, United States). Salinity was analyzed similarly using a 1:5 (w/v) sediment-water suspension measured with a salinity meter (EUTECH SALT6+, Thermo Scientific, United States). The sediment water content was determined by drying 5.0 g fresh sediment at 105 °C to a constant weight. Total carbon (TC) content was determined by elemental analysis (Vario TOC, Elementar, Germany) using 40 mg of homogenized dry sediment (He et al., 2018). Total nitrogen (TN) content were analyzed by an elemental analyzer with 40 mg dry sediment (Vario TOC, Elemental, Germany). The porewater sulfate concentration was determined using ion chromatography (Dionex ICS-600, Thermo Scientific, United States) following the extraction of porewater from 10.0 g of fresh sediment (Sims et al., 1995). Ferrous (Fe^2+^) and ferric (Fe^3+^) iron fractions were sequentially extracted from 0.5 g of fresh sediment according to the protocol described by Cai et al. (2018), and their concentrations were quantified by inductively coupled plasma optical emission spectrometry (ICP-OES; Optima 5300DV, PerkinElmer, United States).

DNA was extracted from fresh sediment samples using a combined approach that incorporated both the traditional freeze-grinding method and the PowerSoil DNA Isolation Kit (Mo Bio Laboratories, Carlsbad, CA, United States) (Zhou et al., 1996), followed by purification. DNA quality was assessed using a NanoDrop One spectrophotometer (Thermo Scientific, United States), with 260/280 and 260/230 absorbance ratios averaging approximately 1.8 and ≥1.7, respectively. Subsequently, DNA concentration was accurately measured using a Qubit 4 Fluorometer (Thermo Scientific, United States) based on fluorescence quantification. The bacterial 16S rRNA gene V3–V4 hypervariable region (expected size: 468 bp) was amplified with universal primers 338F (5′-ACTCCTACGGGAGGCAGCA-3′) and 806R (5′-GGACTACHVGGGTWTCTAAT-3′) (Wang et al., 2020). The amplicon sequencing was conducted using an Illumina HiSeq 2500 platform (Illumina, Inc., CA, United States) with 2 × 250 bp paired-end configuration at Biomarker Technologies Corporation (Beijing, China). Quality filtering and preprocessing of raw sequences were performed using the Linux-based Galaxy pipeline.^1^ First, sequence reads were demultiplexed, and primer sequences were excised using Cutadapt (Martin, 2011). Subsequently, overlapping paired-end sequences were merged, and low-quality sequences (quality score <20) were filtered out via Trimmomatic (Bolger et al., 2014). Potential chimeric sequences were identified and removed from the dataset using UCHIME in reference database mode (Edgar, 2013). High-quality sequences were then clustered into operational taxonomic units (OTUs) at a 97% similarity threshold using UPARSE (Edgar, 2013), with singleton OTUs excluded from downstream analyses. The OTU table was rarefied to an even sequencing depth of 61,207 sequences per sample to eliminate biases arising from differential sequencing efforts before downstream analysis. Taxonomic classification was performed using the Ribosomal Database Project (RDP, Release 11.4) classifier at an 80% confidence threshold (Wang et al., 2007; Zhong et al., 2017).

### 2.4 Statistical analysis

All statistical analyses were performed using R (R Foundation for Statistical Computing, Vienna, Austria) with the vegan, Hmisc, ecodist, and ggplot2 packages, along with the Galaxy pipeline. Microbial alpha diversity was assessed using the Shannon index, calculated from the resampled OTU table for each sample. To evaluate beta diversity patterns, microbial community dissimilarity was examined through principal coordinates analysis (PCoA) based on Bray–Curtis distance matrices. The statistical significance of observed community differences was further verified using multiple approaches, including multiple-response permutation procedure (MRPP), analysis of similarities (ANOSIM), and permutational multivariate analysis of variance (ADONIS).

The statistical analyses, including one-way ANOVA and linear regression, were performed using IBM SPSS 22 (SPSS Inc., United States). One-way ANOVA was employed to compare the sediment parameters, microbial diversity, and microbial abundances among three sampling sites at different depths, as well as to assess the significance of differences in root-respiration CO_2_ fluxes, sediment CO₂ fluxes, sediment temperature, and air temperature across different sampling sites and seasons. The specific *post-hoc* test used Tukey’s HSD for pairwise comparisons following ANOVA. Additionally, Spearman correlation analysis was conducted to examine the potential correlations between environmental factors, CO_2_ flux and microbial community diversity indices or abundances.

Finally, the partial least squares structural equation modeling (PLS-SEM) was applied to fit the data and determine the direct and indirect associations between sediment physicochemical properties, microbial diversity, and sediment CO_2_ flux. All fitting analyses were conducted in R 4.3.2 using the plspm package. To address multicollinearity issues among sediment physicochemical variables, principal component analysis (PCA) was first performed on the sediment properties, and the first two principal components (PC1 and PC2) were used to construct the latent variable “Soil Physicochemical Properties,” which included key sediment indices such as TN, pH, SO_4_^2−^, Fe^3+^, and Fe^2+^. For microbial community diversity, both alpha diversity indices (Shannon, Chao1, Observed OTUs, PD_whole_tree) and β-diversity dispersion (average distance to median) were integrated into the latent variable “Microbial Community.” The significance of the path coefficients was evaluated using the Bootstrap resampling method, with 2,000 bootstrap iterations. The path coefficients were considered significant if the confidence intervals did not contain zero.

## 3 Results

### 3.1 Sediment properties and CO_2_ emissions

Our results demonstrate that sediment properties vary markedly among habitats. Relative to the unvegetated mudflat (pH: 7.76 ± 0.03; salinity: 3.74 ± 0.07 ppt; water content: 31.56 ± 0.63%; TC: 7.86 ± 0.20 g kg^−1^; TN: 0.91 ± 0.05 g kg^−1^), mangrove colonization significantly decreased pH, increased salinity and water content, and enhanced TC and TN concentration accumulation across all depths (*p* < 0.05, Supplementary Figures S1A–E). However, species-specific effects were evident. Sediments of SA exhibited significantly higher pH than KO (*p* < 0.05; Supplementary Figure S1A), but had significant lower salinity (3.90 ± 0.23 ppt), water content (35.30 ± 1.50%), TC content (13.12 ± 1.12 g kg^−1^), and TN content (2.02 ± 0.18 g kg^−1^) compared to KO (salinity: 12.11 ± 0.28 ppt; water content: 48.43 ± 0.87%; TC: 40.4 ± 1.62 g kg^−1^; TN: 4.53 ± 0.09 g kg^−1^) (*p* < 0.05, Supplementary Figures S1B–E). In mangrove habitats, both TC and TN content declined significantly with increasing sediment depth (*p* < 0.05, Supplementary Figures S1D,E).

Sedimentary SO₄^2−^ concentrations followed the order KO (2.88 ± 0.13 g L^−1^) > M (2.37 ± 0.05 g L^−1^) > SA (1.91 ± 0.03 g L^−1^), with a significant difference between SA and M confined to the 0–10 cm layer (*p* < 0.05, Supplementary Figure S1F). Distinct vertical patterns were observed in SO₄^2−^ profiles for KO and SA (*p* < 0.05, Supplementary Figure S1F). For iron valence states in sediments of 0–15 cm depth, Fe^3+^ concentrations ranked KO (7.01–9.78 mg L^−1^) > M (6.13–6.77 mg L^−1^) > SA (4.28–5.88 mg L^−1^). In contrast, Fe^2+^ concentrations in the 0–10 cm layer ranked SA (1.37–1.53 mg L^−1^) > M (1.09–1.46 mg L^−1^) > KO (0.18–0.42 mg L^−1^), with inter-habitat differences most pronounced in surface layers. In deeper layers, Fe^3+^ concentrations at 15–20 cm (KO: 4.84 mg L^−1^; SA: 5.51 mg L^−1^) and Fe^2+^ concentrations at 10–20 cm (KO: 0.86–1.25 mg L^−1^; SA: 1.35–1.49 mg L^−1^) in mangrove communities were significantly lower than those in M (Fe^3+^: 6.37 mg L^−1^; Fe^2+^: 1.68–1.74 mg L^−1^) (*p* < 0.05, Supplementary Figures S1G,H). Depth profiles revealed markedly higher Fe^3+^ concentrations in KO at 0–10 cm compared to 10–20 cm, whereas SA exhibited the opposite trend. Fe^2+^ concentrations increased with depth in M and KO but showed no depth dependence in SA (*p* < 0.05, Supplementary Figures S1G,H).

Sediment temperature exhibited a clear seasonal pattern, with the highest values observed in summer (32.17–34.50 °C) and the lowest in winter (15.70–16.67 °C). Temperatures in spring (24.70–27.00 °C) and autumn (25.93–27.17 °C) showed minimal interseasonal variation.

**Figure 2:**
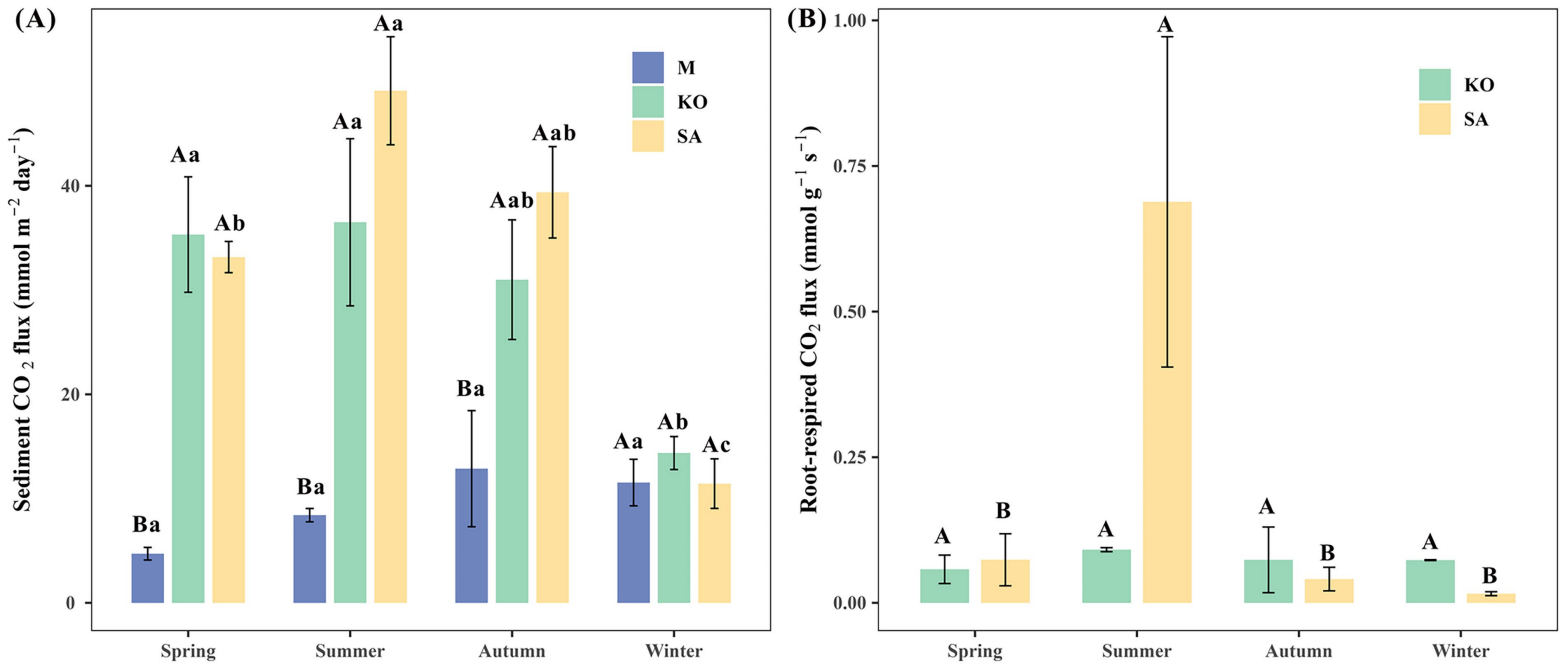
Mean sediment CO_2_ fluxes **(A)** and root-respiration CO_2_ fluxes **(B)** of mudflat (M), *K. obovata* (KO), and *S. apetala* (SA) plantation in different seasons.

Sediment CO₂ fluxes varied markedly across habitats and seasons. With the exception of winter, CO₂ fluxes from mudflat sediments (4.71 ± 0.61 to 12.87 ± 5.57 mmol m^−2^ day^−1^) were significantly lower than those from KO (14.37 ± 1.58 to 36.50 ± 8.02 mmol m^−2^ day^−1^) and SA (11.43 ± 2.38 to 49.12 ± 5.18 mmol m^−2^ day^−1^), whereas no significant difference was observed between KO and SA (*p* < 0.05, Figure 2A). Seasonal variations in CO₂ fluxes were evident in both vegetated habitats. In the SA habitat, CO₂ fluxes exhibited significant seasonal differences (*p* < 0.05, Figure 2A), reaching a maximum in summer, which was approximately 4.3 times higher than in winter. In spring and autumn, CO₂ fluxes in SA averaged 33.17 ± 1.50 mmol m^−2^ day^−1^ and 39.38 ± 4.38 mmol m^−2^ day^−1^, respectively. The CO₂ fluxes at KO exhibited significant seasonal variation (*p* < 0.05, Figure 2A), with relatively high values observed in spring (35.32 ± 5.54 mmol m^−2^ day^−1^) and summer (36.50 ± 8.02 mmol m^−2^ day^−1^), followed by autumn (30.99 ± 5.74 mmol m^−2^ day^−1^), and the lowest values recorded in winter (14.37 ± 1.58 mmol m^−2^ day^−1^).

For root respiration, CO₂ flux in SA exhibited pronounced seasonal dynamics, with a marked peak in summer (0.69 ± 0.28 mmol g^−1^ s^−1^), significantly exceeding fluxes in other seasons (0.015–0.074 mmol g^−1^ s^−1^) and approximately 7.7 times higher than the concurrent KO flux (*p* < 0.05, Figure 2B). In contrast, KO root-respired CO₂ flux showed no significant seasonal variation (*p* > 0.05), remaining within the range of 0.06 ± 0.02 to 0.09 ± 0.01 mmol g^−1^ s^−1^.

### 3.2 Community composition and abundance

**Figure 3:**
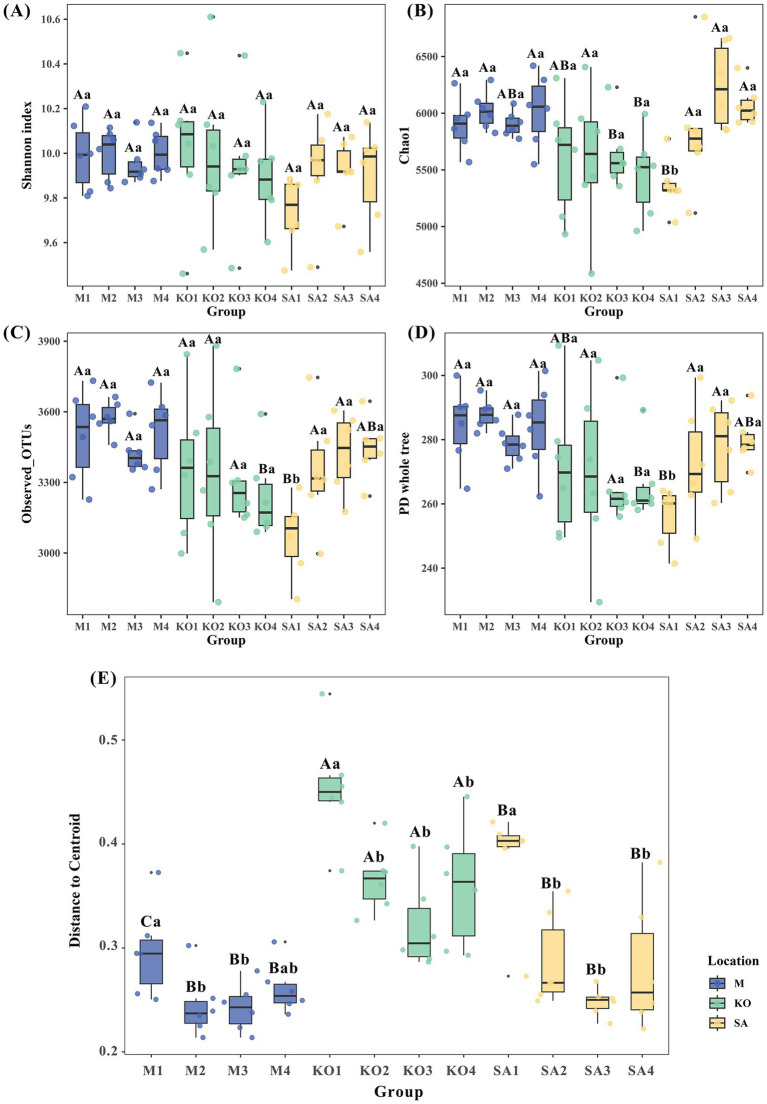
Bacterial α-diversity and β-diversity dispersion (average distance to median) in sediments from mudflat (M), *K. obovata* (KO), and *S. apetala* (SA) habitats. **(A)** Shannon index. **(B)** Chao1 richness. **(C)** Observed OTUs. **(D)** Phylogenetic diversity (PD) whole tree. **(E)** β-diversity dispersion. Different uppercase letters indicate statistically significant differences among the three habitats at the same sediment depth, and different lowercase letters indicate statistically significant differences among sediment depths within the same habitat. (*p* < 0.05, based on one-way ANOVA followed by Tukey’s HSD *post-hoc* test) M1, KO1, and SA1: 0–5 cm depth; M2, KO2, and SA2: 5–10 cm depth; M3, KO3, and SA3: 10–15 cm depth; M4, KO4, and SA4: 15–20 cm depth.

To assess sediment microbial diversity, we evaluated both α-diversity and β-diversity dispersion of bacterial communities across three habitat types. The Shannon index revealed no significant differences among habitats or sediment depths (Figure 3A). However, Chao1 richness in KO sediments exhibited pronounced depth-dependent patterns: at 5–20 cm, values (5,460–5,639) were significantly lower than those in SA (5,841–6,240) and M (5,905–6,023) sediments, whereas at 0–5 cm, KO (5,613) was lower than M (5,899) but higher than SA (5,361) (*p* < 0.05, Figure 3B). Observed OTUs (3,243) and PD whole tree (266) in KO were significantly lower than in SA and M, but only at 15–20 cm depth (*p* < 0.05, Figures 3C,D). Notably, SA sediments exhibited the lowest Chao1, Observed OTUs, and PD values at 0–5 cm depth (*p* < 0.05, Figures 3B–D). Regarding β-diversity, SA (0.38) and M (0.30) differed significantly only in surface sediments (0–5 cm), yet both consistently displayed significantly lower dispersion than KO (0.32–0.45) across all depths. Across all habitats, β-diversity dispersion was highest in the surface layer (0–5 cm) (*p* < 0.05, Figure 3E).

Principal coordinates analysis revealed a clear separation of bacterial communities along the first axis according to habitat type (Supplementary Figure S2), indicating that both mangrove plantation and mangrove species composition significantly influenced the structure of sediment bacterial communities.

Taxonomic profiling of the bacterial communities showed that Proteobacteria (50.9%) was the dominant phylum in mangrove sediments, followed by Chloroflexi (19.2%), Bacteroidota (5.1%), Actinobacteriota (4.0%), and Cyanobacteria (2.4%) (Supplementary Figure S3A). At the genus level, many of the relatively abundant taxa were associated with sulfur cycling. *Thioprofundum* (3.4%) was the most abundant genus, followed by *Litorilinea* (2.0%), *Sulfurimonas* (1.9%), *Sulfurovum* (1.4%), *Longilinea* (1.3%), *Desulfobulbus* (1.2%), *Desulfovirga* (1.0%), *Desulfosarcina* (1.0%), and *Thermomarinilinea* (1.0%) (Supplementary Figure S3B).

**Figure 4:**
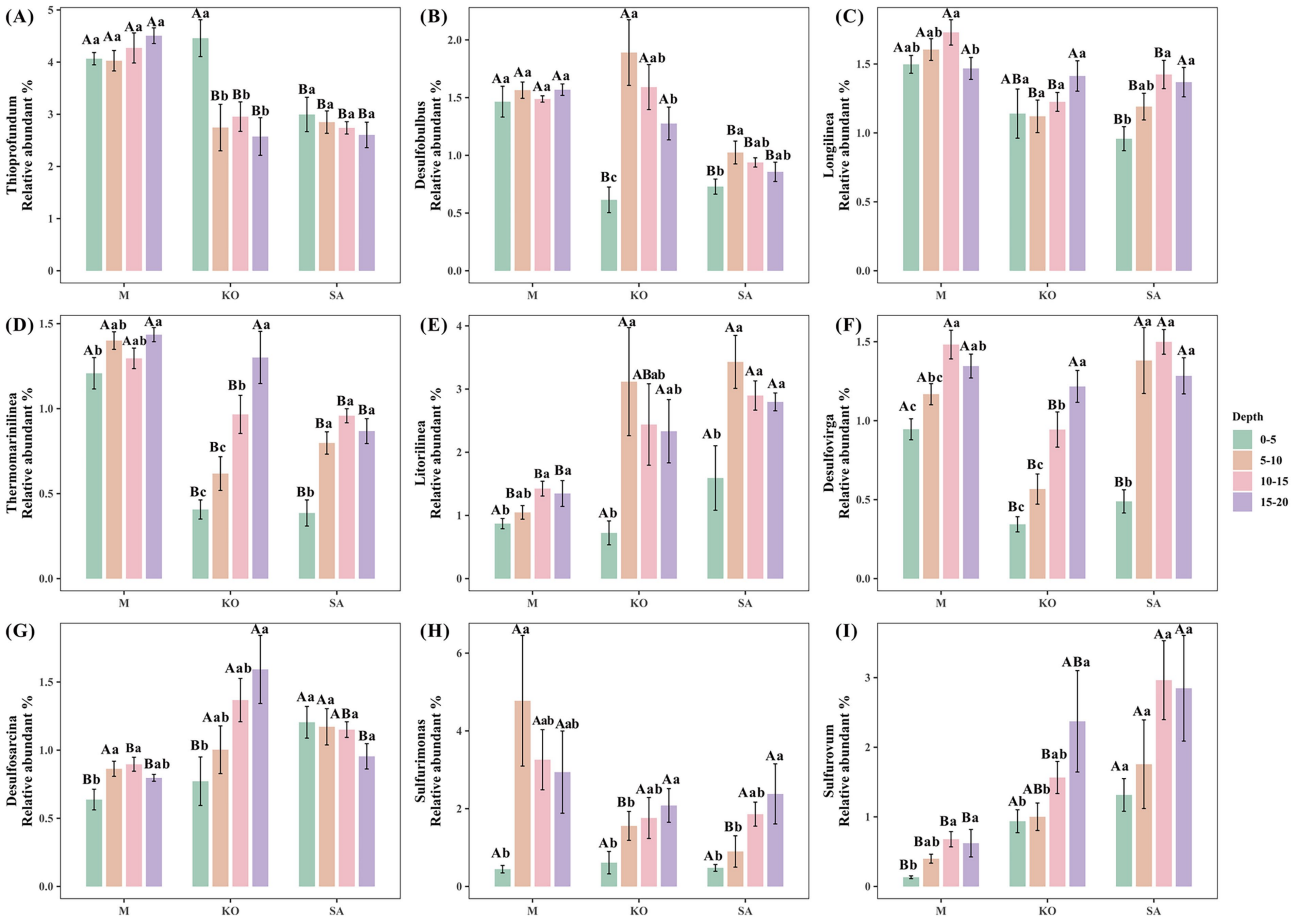
Different relative abundances **(A–I)** of dominant bacterial genera. Values are presented as mean ± standard error (*n* = 6).

Mangrove plantation significantly altered the composition of sediment bacterial communities. In unvegetated mudflats, the relative abundances of *Thioprofundum* (4.2%), *Longilinea* (1.6%), and *Thermomarinilinea* (1.3%) were significantly higher compared with mangrove-vegetated sites, whereas *Litorilinea* (1.2%) and *Sulfurovum* (0.5%) were significantly lower (*p* < 0.05, Figure 4). Comparison between the two mangrove species revealed that *Sulfurovum* (2.2%) and *Desulfovirga* (1.2%) were substantially more abundant in SA sediments, whereas *Desulfobulbus* (0.9%) was significantly less abundant compared with KO sediments (*p* < 0.05, Figure 4). Depth profiles showed that across all three habitats, *Litorilinea* (0.7–1.6%) and *Sulfurimonas* (0.4–0.6%) consistently exhibited lower relative abundances in the surface layer (0–5 cm) than in deeper layers. In KO sediments, the relative abundances of the sulfate-reducing genera *Desulfovirga* and *Desulfosarcina*, as well as the sulfur-oxidizing genus *Sulfurovum*, increased significantly with sediment depth (*p* < 0.05, Figure 4).

**Figure 5:**
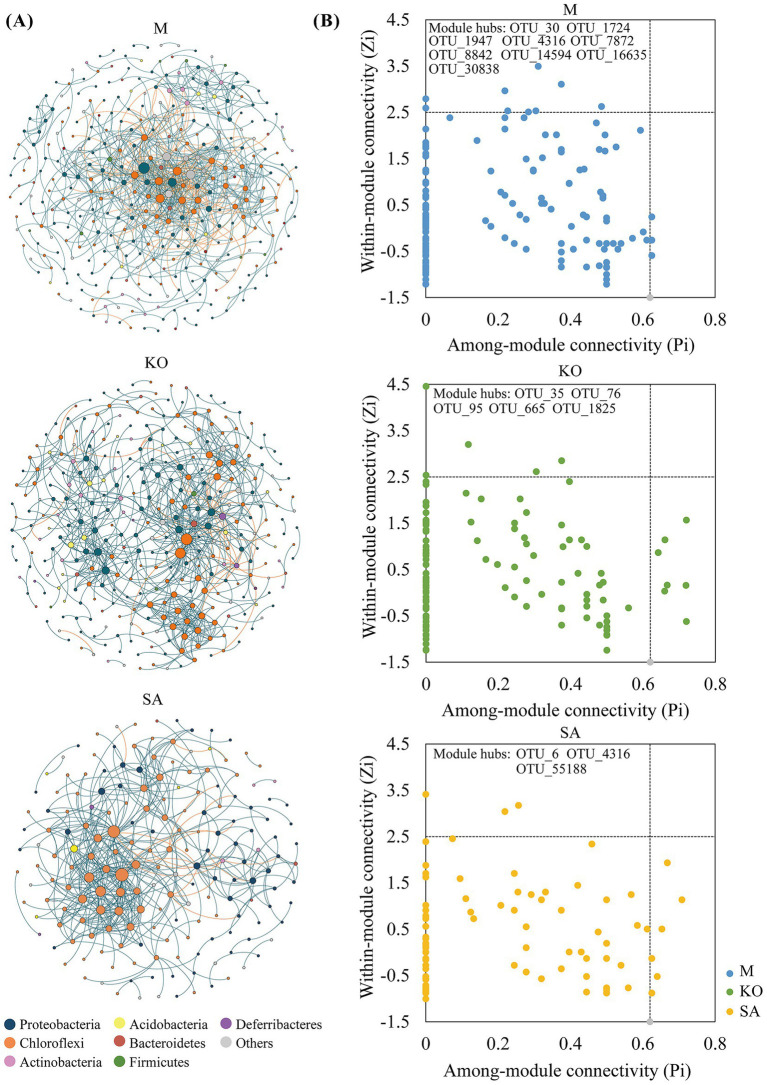
Bacterial community networks in three habitats **(A)**, and distribution of network nodes based on within-module connectivity (Zi) and among-module connectivity (Pi) **(B)**.

To investigate potential microbial interactions in mangrove sediments, we constructed molecular ecological networks. The bacterial co-occurrence network in site M exhibited the greatest number of nodes and edges, whereas the SA sediment network contained the fewest. Compared with the KO bacterial network, the SA network exhibited greater complexity, as evidenced by a significantly shorter average path length (SA: 3.519 vs. KO: 5.615), a higher average clustering coefficient (SA: 0.305 vs. KO: 0.240), and increased average connectivity (SA: 5.227 vs. KO: 4.406) (Figure 5A and Table 1), collectively indicating stronger microbial interactions. In contrast, the KO network had a higher modularity value, suggesting that although overall node-to-node interactions were weaker, intra-module connections were more pronounced. Taxonomic composition analysis revealed that nodes in the KO network were predominantly affiliated with the phyla Proteobacteria and Chloroflexi. In contrast, the relative abundance of Chloroflexi was notably higher in the SA network (Figure 5A).

**Table 1:** Topological properties of microbial networks in mudflat and mangrove sediment samples (mean ± 1SE, *n* = 6).

Network type	Topological properties	M	KO	SA
Empirical network	Nodes	348	325	181
	Edges	831	716	473
	*R*^2^ of power law	0.88	0.912	0.898
	Average path	4.535	5.615	3.519
	Average clustering coefficient	0.22	0.24	0.305
	Average connectivity	4.776	4.406	5.227
	Modularity (module number)	0.572 (43)	0.675 (27)	0.526 (18)
Random network	Average path	3.498 ± 0.045	3.713 ± 0.041	3.118 ± 0.042
	Average clustering coefficient	0.054 ± 0.007	0.030 ± 0.005	0.097 ± 0.013
	Modularity	0.416 ± 0.006	0.455 ± 0.006	0.368 ± 0.007

M, KO and SA were represented as mudflat, *K. obovata* and *S. apetala*, respectively.

To assess the topological roles of network nodes, we classified them based on within-module connectivity (Zi) and among-module connectivity (Pi) metrics. In total, 17 module hubs were identified, including 9 in M, 5 in KO, and 3 in SA (Figure 5B). The module hubs of M derived from Proteobacteria (OUT_30, OUT_1724, OUT_7872, OUT_14594, OUT_30838) and Chloroflexi (OUT_4316, OTU_16635), KO derived from Proteobacteria (OUT_35, OUT_95) and Chloroflexi (OUT_76, OTU_665, OTU_1825), SA derived from Proteobacteria (OUT_6) and Chloroflexi (OUT_4316, OTU_55188).

### 3.3 Correlation between biotic, abiotic factors and CO_2_ fluxes

**Figure 6:**
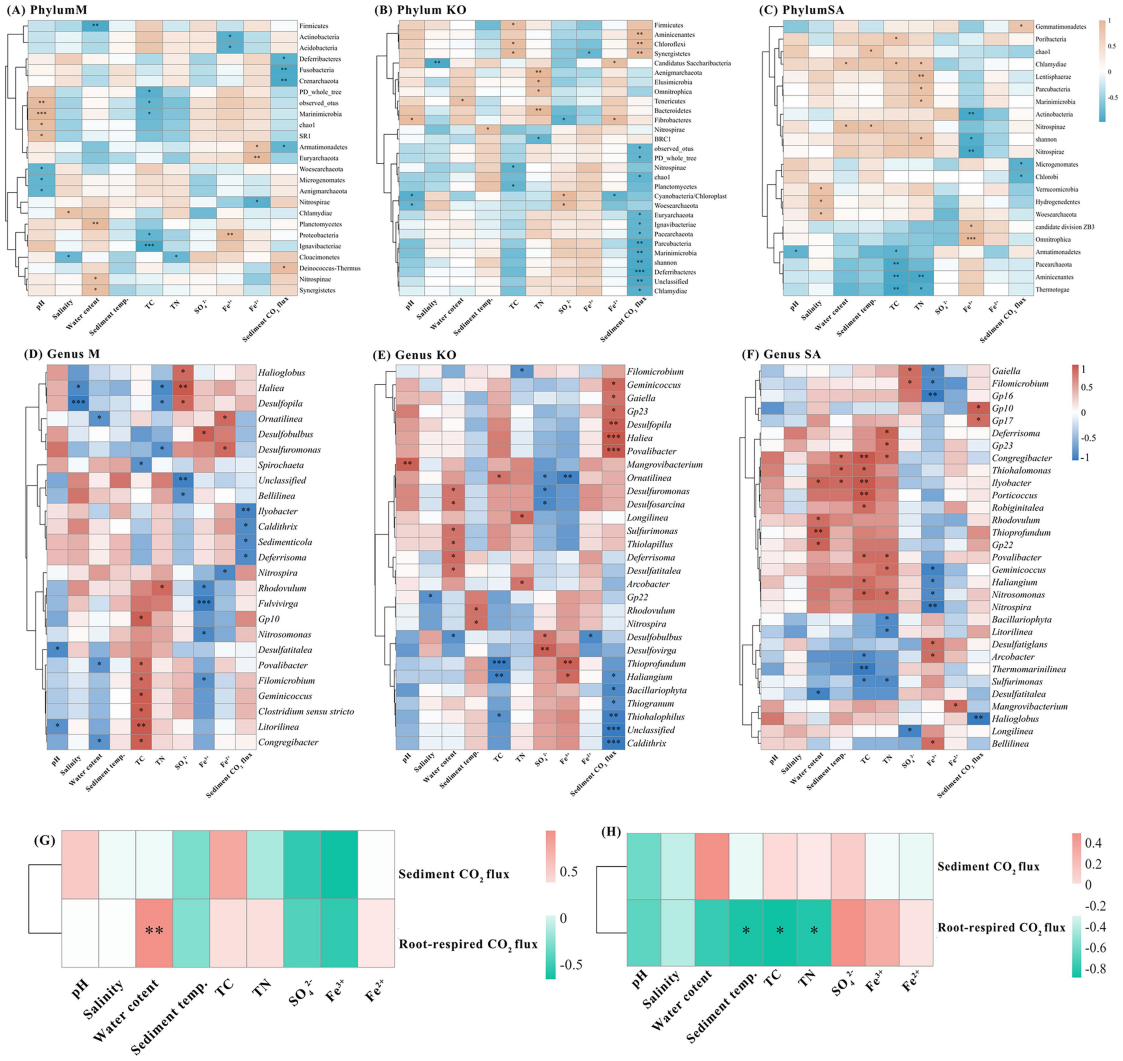
Correlations between dominant sediment bacterial taxa (phylum level: **A–C**, genus level: **D–F**) and sediment environmental factors as well as sediment CO_2_ flux. Correlations between sediment environmental factors and sediment CO_2_ flux (**G–H**).

To explore the interrelationships among sediment physicochemical properties, CO₂ emission fluxes, and microbial communities, we generated a heatmap based on Spearman correlation analysis (Figures 6A–C). In the M habitat, bacterial α-diversity exhibited a significant negative correlation with TC content (*p* < 0.05), but showed a significant positive correlation with pH (*p* < 0.01). In contrast, in the KO habitat, microbial α-diversity was negatively associated only with sediment CO₂ emission flux (*p* < 0.01). For the SA habitat, both Shannon and Chao1 indices increased significantly with rising TN content and sediment temperature, but decreased with higher Fe^3+^ concentrations (*p* < 0.05).

We further examined the correlations between phylum-level microbial groups, environmental parameters, and sediment CO₂ flux, retaining only statistically significant associations (Figures 6A–C). In the M habitat, pH showed a highly significant positive correlation with Marinimicrobia (*p* < 0.001). The relative abundance of Firmicutes was significantly reduced with increasing water content but showed a positive association with Planctomycetes (*p* < 0.01). TC content was negatively correlated with Proteobacteria (*p* < 0.05). Fe^2+^ concentration was positively correlated with Euryarchaeota (*p* < 0.01), whereas Fe^3+^ concentration exhibited a significant positive correlation with Proteobacteria (*p* < 0.01) but significant negative correlations with Actinobacteria and Acidobacteria (*p* < 0.05). In the KO habitat, TC content was positively associated with Chloroflexi and Firmicutes (*p* < 0.05). Bacteroidetes abundance increased significantly with rising TN concentration (*p* < 0.05). Sediment CO₂ flux was positively correlated with Aminicenantes, Chloroflexi, and Synergistetes, while negatively correlated with Parcubacteria, Marinimicrobia, and Deferribacteres (*p* < 0.01). In the SA habitat, TC content was negatively correlated with Aminicenantes and Thermotogae (*p* < 0.01). Similarly, TN content was negatively associated with Aminicenantes (*p* < 0.01). Moreover, Fe^3+^ concentration was negatively correlated with Actinobacteria and Nitrospirae (*p* < 0.01).

For genus-level microorganisms, we selected the 50 most abundant genera for correlation analysis and retained only statistically significant associations (Figures 6D–F). In the M habitat, salinity and nutrient factors were the main drivers, showing strong negative correlations with *Desulfopila*and *Haliea*, while TN concentration and iron species were selectively linked with sulfate-reducing genera. Sediment CO₂ flux was negatively correlated with *Ilyobacter*. In the KO habitat, water content strongly promoted several sulfate-reducing and sulfur-oxidizing genera (Desulfuromonas, Desulfosarcina, Sulfurimonas, Thiolapillus, Deferrisoma, and Desulfatitalea), while TC and TN content exerted opposite influences on different taxa. Iron species also showed contrasting associations, with Fe^3+^ concentration positively correlated with *Thioprofundum* and Fe^2+^ concentration negatively with *Desulfobulbus* (*p* < 0.05). Sediment CO₂ flux increased with *Haliea* and *Desulfopila* but decreased with *Caldithrix* and *Thiohalophilus* (*p* < 0.001). In the SA habitat, water content and TC content were positively associated with sulfur-related genera such as *Thioprofundum* and *Thiohalomonas* (*p* < 0.05), while TN and SO₄^2−^ concentrations exhibited negative correlations with several anaerobic lineages (*p* < 0.05). Fe^3+^ concentration was positively linked to *Nitrospira* (*p* < 0.05), and sediment CO₂ flux decreased with the abundance of *Halioglobus* (*p* < 0.01).

In addition, we also analyzed the relationship between sediment CO_2_ flux, root-respired CO_2_ flux, and sediment physical and chemical properties (Figures 6G,H). KO root-respired CO_2_ flux was significantly positively correlated with sediment water content (*p* < 0.01), while SA was significantly negatively correlated with sediment temperature, TC, and TN (*p* < 0.05).

Partial Least Squares Structural Equation Modeling (PLS-SEM) was employed to elucidate the relationships among sediment physicochemical properties, microbial community diversity, and sediment CO₂ flux in mangrove ecosystems (Supplementary Figure S4). The results demonstrated that sediment physicochemical properties exerted a strong and significant influence on microbial community diversity, with a path coefficient of −0.710, underscoring the pivotal role of soil characteristics in regulating microbial diversity within mangrove sediments.

With respect to sediment CO₂ flux, sediment physicochemical properties influenced respiration through both a direct and an indirect pathway. The direct effect was strong and significant (path coefficient = 0.691). In addition, an indirect effect was observed, whereby soil physicochemical properties affected microbial community diversity, which in turn influenced sediment CO₂ flux (indirect effect = 0.206). Taken together, the total effect of soil on sediment CO₂ flux reached 0.897, indicating that both direct regulation by soil properties and mediation through microbial communities are critical mechanisms driving CO₂ emission dynamics. The model explained 50.4% of the variance in microbial community diversity (*R*^2^ = 0.504) and 84.7% of the variance in sediment CO₂ flux (*R*^2^ = 0.847). In addition, the overall goodness of fit (GOF) index reached 0.602, indicating satisfactory explanatory power and validity.

In addition, the present study’s results indicated that mangrove tree species significantly shape sediment physicochemical conditions. Building on this evidence, it is plausible to infer that tree species may indirectly affect microbial diversity by altering sediment properties, which subsequently influences sediment CO₂ fluxes dynamics. This finding emphasizes the intricate interplay between biotic and abiotic drivers in mangrove ecosystems and reinforces the need to consider both vegetation and edaphic factors when evaluating carbon cycling processes.

## 4 Discussion

### 4.1 Species-specific mangrove afforestation differentially modulates sediment physicochemistry and below-ground CO₂ effluxes

The development of mangrove root systems promotes the secretion of root exudates and the decomposition of litter, both of which generate substantial amounts of organic acids (Guan et al., 2018). These acids lower the sediment pH and contribute to the accumulation of total carbon and total nitrogen (Ono et al., 2015; Van Do et al., 2015; Guan et al., 2018; Hu et al., 2024). In addition, mangrove plants excrete excess salts and accumulate them in the sediment through their roots and a small amount of litter from branches and leaves, thereby increasing sediment salinity. This process leads to persistently elevated salinity levels across sediment profiles (Breithaupt et al., 2018), a phenomenon observed in the different mangrove communities examined in this study. However, variations in litter decomposition rates, root turnover, and the composition of root exudates among mangrove species strongly influence the accumulation of sediment organic matter. Slow decomposition tends to promote the accumulation of organic matter and nutrients, whereas rapid decomposition accelerates nutrient cycling and enhances plant uptake (Lima and Colpo, 2014). Our previous research demonstrated that the root dynamics of *S. apetala* and *K. obovata* exert substantial effects on sediment carbon accumulation (Isaac and Achuthan Nair, 2005; He et al., 2021). In addition, root-derived exudates can interact with clay minerals as well as OH-Al and OH-Fe complexes, promoting the formation of large aggregates that improve sediment structural stability (Violante and Caporale, 2015). Demonstrated that root exudates from *Bruguiera gymnorrhiza* enhance the labile organic matter content and stimulate microbial activity in the rhizosphere. Consistent with this perspective, our study revealed pronounced species-specific differences in sediment sulfur and iron cycling. Specifically, SO₄^2−^ and Fe^3+^ concentrations in *K. obovata* sediments were significantly higher than those in *S. apetala*, whereas Fe^2+^ concentrations were comparatively lower. The elevated organic carbon content in *K. obovata* sediments likely provides abundant electron donors for sulfate-reducing bacteria (SRB), thereby facilitating intensified sulfate reduction and contributing to higher sulfate concentrations (Jørgensen, 2003; Taketani et al., 2010). During sulfate reduction, the produced H₂S reacts with Fe^3+^, reducing it to Fe^2+^ and forming iron sulfides such as FeS and FeS₂ (Sherman and Howarth, 1998). This process leads to relatively higher Fe^3+^ concentrations and lower Fe^2+^ concentrations in *K. obovata* sediment in the present study. With increasing depth, hypoxic conditions intensify, making Fe^2+^ less susceptible to oxidation, while Fe^3+^ declines due to ongoing reduction processes. In the deeper layers with diminished organic matter availability, sulfate reduction weakens, resulting in elevated SO₄^2−^ concentrations (Kristensen et al., 2011). Mangrove respiratory roots deliver oxygen to surface sediments, fostering the accumulation of sulfur-oxidizing bacteria (SOB) in the upper layers (Okello et al., 2020). The relatively high pH of sediments in *S. apetala* plantation created a more neutral environment, in which the oxidation end-products are SO₄^2−^ rather than S₄O₆^2−^ (Kitaya et al., 2002; Frigaard and Dahl, 2009). With depth, the abundance of SOB progressively decreases, while sulfate-reducing bacteria (SRB) activity intensifies, resulting in vertical stratification of SO₄^2−^ and Fe^3+^ concentrations in sediments.

CO₂ fluxes from mangrove sediments are regulated by a complex interplay of biotic and abiotic factors, including climate, mangrove species, sediment carbon content, tidal position, and the activity of microorganisms and biofilms at the sediment surface (Leopold et al., 2013; Kristensen et al., 2017). Among these, temperature is recognized as a major driver of seasonal variability in subtropical regions (Chen L. et al., 2012; Livesley and Andrusiak, 2012), with elevated sediment temperatures promoting microbial activity and accelerating organic matter mineralization (Lovelock, 2008; Sun et al., 2024). In the present study, carbon accumulation induced by mangrove planting resulted in significantly greater sediment CO₂ fluxes in *S. apetala* and *K. obovata* sites compared with the mudflat. Moreover, sediment CO₂ fluxes exhibited pronounced seasonal fluctuations, with *S. apetala* releasing ~4.3 times more CO₂ in summer than in winter, while *K. obovata* also showed elevated summer fluxes, in agreement with previous findings (Hien et al., 2018). Notably, root respiration in both *S. apetala* and *K. obovata* was at its highest level during summer, highlighting that belowground root activity is an important contributor to sediment CO₂ fluxes during this season. This phenomenon likely reflects their contrasting life-history strategies and origins. As a fast-growing exotic species, *S. apetala* may have a higher metabolic demand for carbon and nutrients during the warm summer growing season to support its rapid biomass accumulation (Liu et al., 2014; Wang C. et al., 2022; Wang Y. et al., 2022). This could drive increased respiratory activity in its fine roots. In contrast, the native *K. obovata*, which tends towards slower growth, may exhibit a more conservative and stable respiratory strategy across seasons. Furthermore, this divergence could also be attributed to differential thermal adaptation; having evolved in local conditions, *K. obovata* might possess better-regulated physiological processes that are less sensitive to temperature fluctuations, whereas the physiological processes of the introduced *S. apetala* might be more thermally responsive, leading to higher respiratory activity during peak temperatures (Chen L. et al., 2012; Liu et al., 2019). This interplay between growth strategy and thermal adaptation underscores how species identity shapes not only carbon storage but also the temporal pattern of carbon release belowground.

These findings emphasize that mangrove restoration and species selection can significantly alter sediment carbon dynamics. Seasonal shifts in microbial and root-driven respiration should therefore be incorporated into regional and global carbon budget assessments, as neglecting such variability may underestimate the contribution of mangrove sediments to atmospheric CO₂ fluxes. This highlights the dual role of mangroves as both critical carbon sinks and dynamic sources, with implications for climate mitigation strategies. It is important to note that our measurements, while capturing seasonal variation, represent snapshots in time. Sediment CO₂ fluxes are also influenced by short-term factors such as tidal inundation and diel cycles. Therefore, our estimates may not capture the full variability of these dynamic ecosystems. Future studies incorporating high-frequency, long-term monitoring across multiple tidal conditions will be valuable to refine our annual carbon budget estimates and improve their generalizability.

### 4.2 Microbial community assembly driven by mangrove zonation and species identity

The Chao1 index revealed that different mangrove species exert stratified effects on microbial diversity. In the *S. apetala* habitat, microbial richness was higher in the 5–20 cm sediment layer, whereas in the *K. obovata* habitat, greater richness was observed in the 0–5 cm layer. β-diversity indices and principal coordinate analysis further indicated stronger spatial heterogeneity in the microbial community structure of *K. obovata*. Overall, mangrove afforestation and species composition were identified as key drivers of sediment microbial community differentiation, in agreement with previous findings (Duan et al., 2025; Li et al., 2021). In the present study, Chloroflexi and Proteobacteria dominated the sediment communities, accounting for ~70% of the abundance, and were also the main taxa forming key nodes in the molecular ecological networks of both habitats. Given their high sensitivity to ecological variation, these two phylum likely underpin community compositional shifts in response to sediment environmental changes (Wang C. et al., 2022; Wang Y. et al., 2022). At the genus level, we found that the most abundant taxa were primarily involved in sulfur cycling and anaerobic fermentation, including sulfur-oxidizing bacteria, sulfate-reducing bacteria, and anaerobic fermenters. In mudflat sediments, periodic tidal flushing and oxygen exposure facilitated the formation of an oxidative surface layer favorable to SOBs (Røy et al., 2007), which explaining the higher abundance of *Thioprofundum* relative to the two mangrove sediments in this study. Meanwhile, rapid accumulation and decomposition of organic matter derived from microplankton biofilms (e.g., extracellular polymeric substances) in mudflats supported abundant fermenters (Mckew et al., 2013). This explains the higher abundance of anaerobic fermenters in the mudflat sediment. Species identity further shaped community composition. In *K. obovata* sediments, the abundance of *Desulfobulbus* was significantly higher, while *Desulfovirga* was lower than in *S. apetala*. These patterns correlated with higher sulfate and Fe^3+^ concentrations in *K. obovata*. Depth-specific differences were also observed, with *Desulfosarcina* having a relatively low abundance and varying significantly only at depths of 10–20 cm. Metabolic specialization further explained these patterns: *Desulfobulbus* preferentially reduces simple substrates such as ethanol, acetate, and lactate, while *Desulfosarcina* utilizes aromatic compounds, suggesting that sulfur reduction in these habitats is largely driven by small-molecule carbon inputs (Kleindienst et al., 2014; van Houten et al., 2015). The enrichment of *Sulfurovum* in *S. apetala* sediment aligned with the earlier observation of environmental factors and SOB abundance. Vertical distribution patterns also highlighted functional partitioning: both SRBs and SOBs increased with depth in *K. obovata* sediment, indicating sulfur cycle coupling reactions dominate in the anoxic zone. SOBs oxidize SRB-derived H₂S, a coupling mechanism that also explains the elevated Fe^2+^ concentrations in deep *K. obovata* layers (Hai et al., 2016; Li et al., 2021).

Molecular ecological network analysis provided additional insights into interspecies interactions. The *S. apetala* network exhibited higher average connectivity (5.227) and shorter path length (3.519), suggesting a more efficient community response mechanism that enhances adaptability to environmental fluctuations (Yuan et al., 2021). In addition, nodes affiliated with Chloroflexi were markedly enriched in *S. apetala*, a phylum containing diverse organic matter degraders. This enrichment may contribute to its higher and more seasonal variable sediment CO₂ flux (Bayer et al., 2018; Colatriano et al., 2018). By contrast, the *K. obovata* network displayed a higher degree of modularity, indicating a structure composed of more distinct, tightly-knit modules, which may reflect functional specialization (Alcalá-Corona et al., 2021). Among its 17 key nodes, 65% belonged to the Proteobacteria phylum, suggesting a central role in regulating sulfate reduction and influencing carbon mineralization efficiency.

### 4.3 Sediment-microbe interactions link species effects to carbon flux regulation

The PLS model demonstrates that tree species identity significantly reshapes microbial community structure and functional partitioning by altering sediment physicochemical properties, thereby driving differences in sediment CO_2_ fluxes. In the *K. obovata* plantation, CO₂ emission fluxes were significantly associated with a wide range of microbial taxa (15 at the phylum level and 12 at the genus level), whereas in the *S. apetala* plantation, fluxes correlated with only 3–4 microbial taxa. This contrast suggests that *K. obovata* sediment, regulated by multiple microbial groups, maintains greater stability under environmental fluctuations, which may explain their lower sensitivity to seasonal variation. In contrast, SOBs appear more susceptible to environmental changes than SRBs, and the higher SOBs abundance in *S. apetala* sediments may contribute to the elevated summer CO₂ fluxes observed in this habitat (Li et al., 2021). Organic matter decomposition in mangroves is largely driven by fermentation and sulfate reduction processes mediated by SRBs (Kristensen et al., 1991), which account for more than 50% of organic carbon degradation (Jørgensen, 2003). Consequently, sediment CO₂ fluxes in mangroves are strongly linked to the diversity and abundance of SRB taxa. In *K. obovata* sediment, higher TC and TN contents promote the growth of Chloroflexi, Firmicutes, and Bacteroidetes, which produce CO₂ through anaerobic fermentation. Simultaneously, water and carbon contents stimulate sulfate-reducing taxa associated with sulfur cycling (Hien et al., 2018). In *S. apetala* sediment, microbial α-diversity increases with TN and temperature but is inhibited by Fe^3+^. Previous studies indicate that iron availability influences sulfate reduction, with excessive Fe^3+^ concentrations even suppressing SRB activity (Attri et al., 2011). Moreover, water content and TC favor the proliferation of thiometabolizing genera such as *Thioprofundum* and *Thiohalomonas*, while TN and SO₄^2−^ constrain anaerobic fermentation. Differences in the proportion of recalcitrant organic matter (e.g., lignin) in litter between *S. apetala* and *K. obovata* may also underlie variation in carbon utilization strategies. In *S. apetala* sediment, microbial communities tend to adopt an R-strategy, allocating more carbon to protein synthesis (Lu et al., 2024). This metabolic preference increases the contribution of microbial residue carbon to the sediment carbon pool, which may accelerate the mineralization of active carbon fractions and enhance CO₂ emission potential (Lu et al., 2024). Our findings suggested that although overall CO₂ emission rates do not differ significantly between mangrove species, the underlying microbial mechanisms of CO₂ production exhibit habitat-specific patterns shaped by sedimentary conditions.

The sediment–microbe interactions not only clarify species-specific differences in CO₂ emission mechanisms but also demonstrate how tree species influence carbon fluxes through the regulation of benthic microbial communities. This process is critical for mangrove carbon cycling: on the one hand, mangrove species alter sediment physicochemical properties, thereby indirectly shaping microbial functional partitioning and determining the pathways of organic matter decomposition and CO₂ release; on the other hand, such bottom-up regulation governs the balance between carbon sink and source functions of mangroves under environmental fluctuations. More broadly, the coupling of sediment properties, microbial communities, and carbon fluxes in mangroves provides key insights into regional carbon budgets and offers valuable implications for understanding and mitigating global carbon cycle dynamics and climate change.

Finally, we acknowledge a limitation of this study: the sediment physicochemical and microbial community data were obtained from a single sampling time (spring 2018). While these data robustly reflect the fundamental differences between habitats, future research incorporating seasonal sampling of sediments would provide a more comprehensive understanding of the temporal dynamics linking microbial communities and carbon fluxes.

## 5 Conclusion

This study demonstrates that mangrove species identity exerts a critical influence on sediment CO₂ fluxes by altering sediment physicochemical properties and shaping microbial community structure. Mangrove colonization significantly enhanced sediment salinity, water content, and carbon concentration, thereby increasing sediment CO₂ fluxes compared with unvegetated mudflats. Seasonal variations were pronounced, with *S. apetala* exhibiting markedly higher CO₂ fluxes in summer, driven by both sediment CO₂ fluxes and root-respiration CO_2_ fluxes, while *K. obovata* maintained relative stable fluxes across seasons. Importantly, *K. obovata* combined a higher carbon storage capacity with more stable CO₂ emission dynamics, suggesting that native species may be preferable in restoration projects that prioritize long-term carbon sequestration. Microbial communities were dominated by Proteobacteria and Chloroflexi, but their organization differed between species. *S. apetala* sediments supported tighter microbial interactions, whereas *K. obovata* exhibited higher modularity and functional specialization. PLS-SEM analysis further revealed that sediment properties strongly constrained microbial diversity and regulated CO₂ flux through both direct and indirect pathways. These findings underscore that sediment–microbe interactions are central to carbon flux regulation and that tree species modulate these processes in distinct ways. The present study highlights the dual role of mangrove ecosystems as both carbon sinks and carbon sources, with species-specific differences in root-respiration and sediment CO₂ fluxes shaping their contribution to the carbon cycle. Recognizing these mechanisms is essential for accurate carbon budget assessments and for guiding mangrove restoration strategies. Future work should integrate long-term monitoring and multi-species comparisons to better capture the temporal and spatial variability of mangrove carbon dynamics under changing environmental conditions.

## Data Availability

The datasets presented in this study can be found in online repositories. The names of the repository/repositories and accession number(s) can be found in the article/Supplementary material.

## Footnotes

^1^ https://usegalaxy.cn/
