# Supplementary material for: Species-specific difference of CO₂ emissions in mangroves: coupling sediment physicochemistry and microbial communities
Source: Front Microbiol. 2025 Oct 29;16:1694593. doi: 10.3389/fmicb.2025.1694593 (PMC12608084; doi:10.3389/fmicb.2025.1694593)
Supplement: Supplementary file 1 [file Data_Sheet_1.zip › Supplementary_Figures_S1-S4.docx]

**Supplementary Figure**

**Species-Specific Difference of CO₂ Emissions in Mangroves: Coupling Sediment Physicochemistry and Microbial Communities**

Ziying He^1†^, Xueyin Zhuang^2†^, Jin Liang^2,3*^, Yisheng Peng^2*^, Huaye Sun^4^, Zhushi Yin^2^, Meng Xia^1^, Lili Zhao^2^, Bowen Hu^2,5^, Ming Qu^1^, Weidong Zhu^1^

Supplementary FIGURE S1

Supplementary FIGURE S2

Supplementary FIGURE S3

Supplementary FIGURE S4


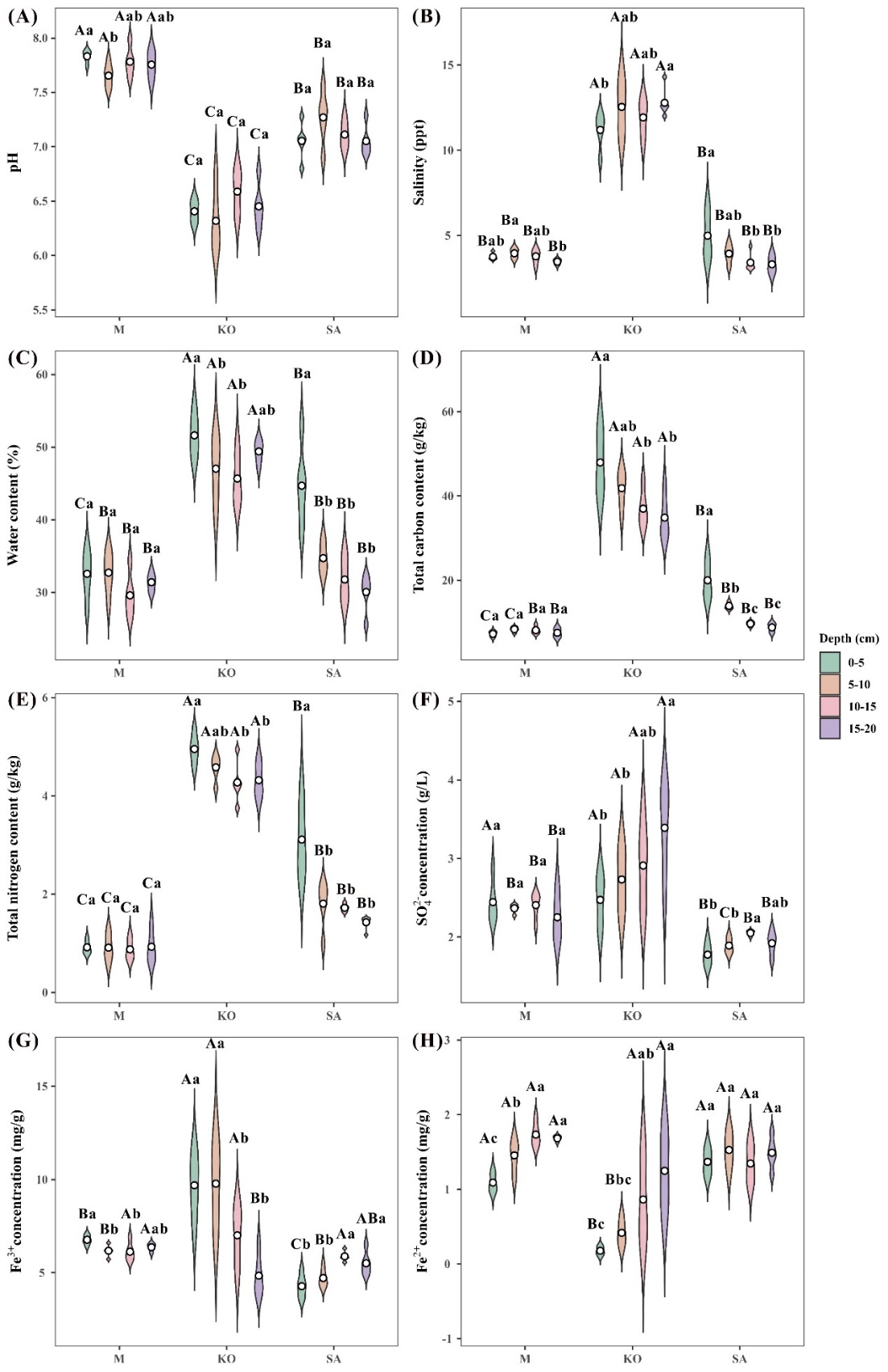


FIGURE S1 Sediment physicochemical properties of mudflat (M), *K. obovata* (KO), and *S. apetala* (SA) plantation. Error bars represent 1SE (n=5). Different uppercase letters indicate significant differences among habitats at the same depth, different lowercase letters denote significant differences among different depths within the same habitat (*p*< 0.05, based on one-way ANOVA followed by Tukey's HSD post hoc test)


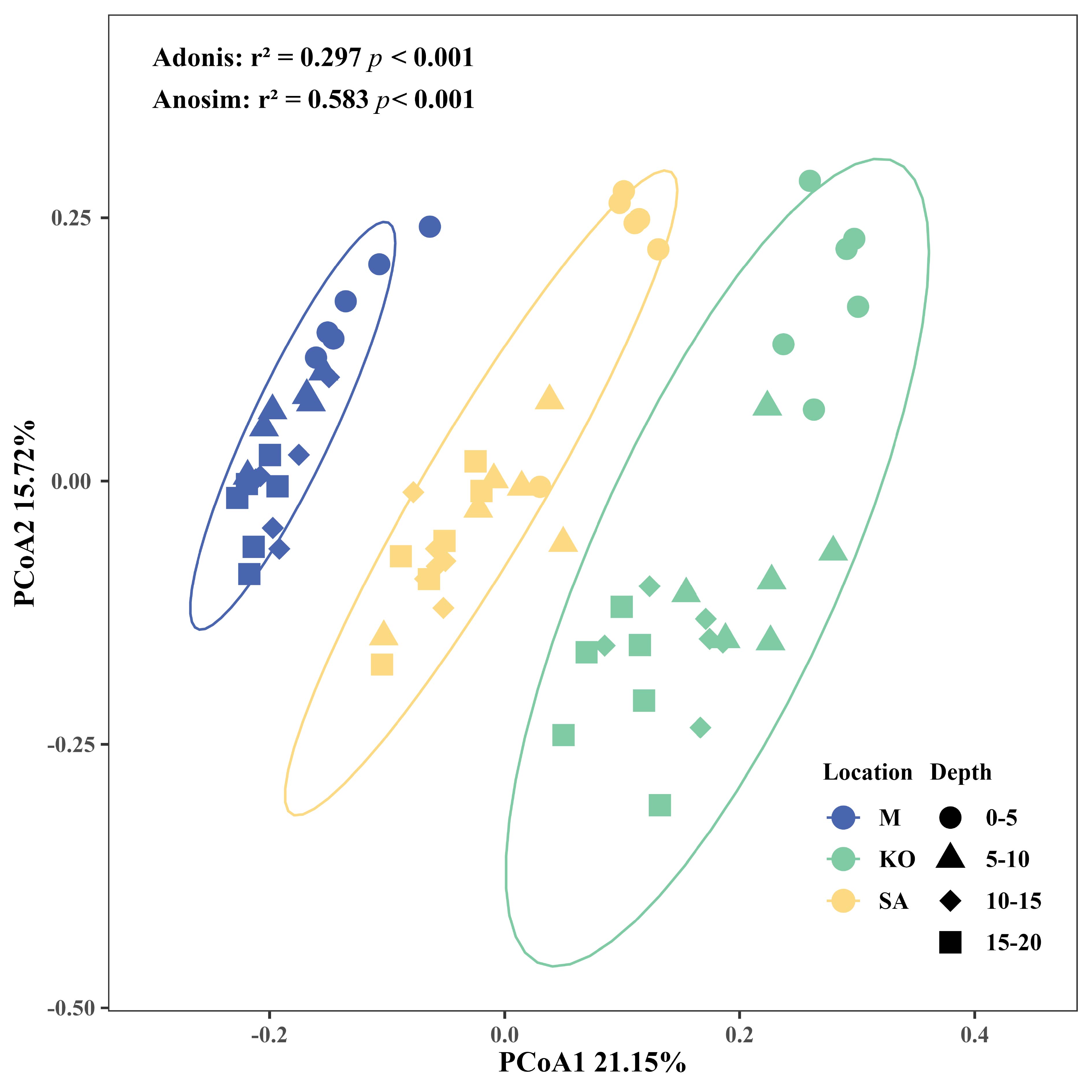


FIGURE S2 Principal coordinates analysis (PCoA) of bacterial community structures. Ellipses indicate the 95% confidence interval for samples within the same group. The percentages following the PCoA1 and PCoA2 axes represent the proportion of variation explained by each axis. Colors and shapes denote different habitats and depths, respectively. Community dissimilarities were assessed using Adonis and ANOSIM tests. M: mudflat; KO: *K. obovata*; SA: *S. apetala*


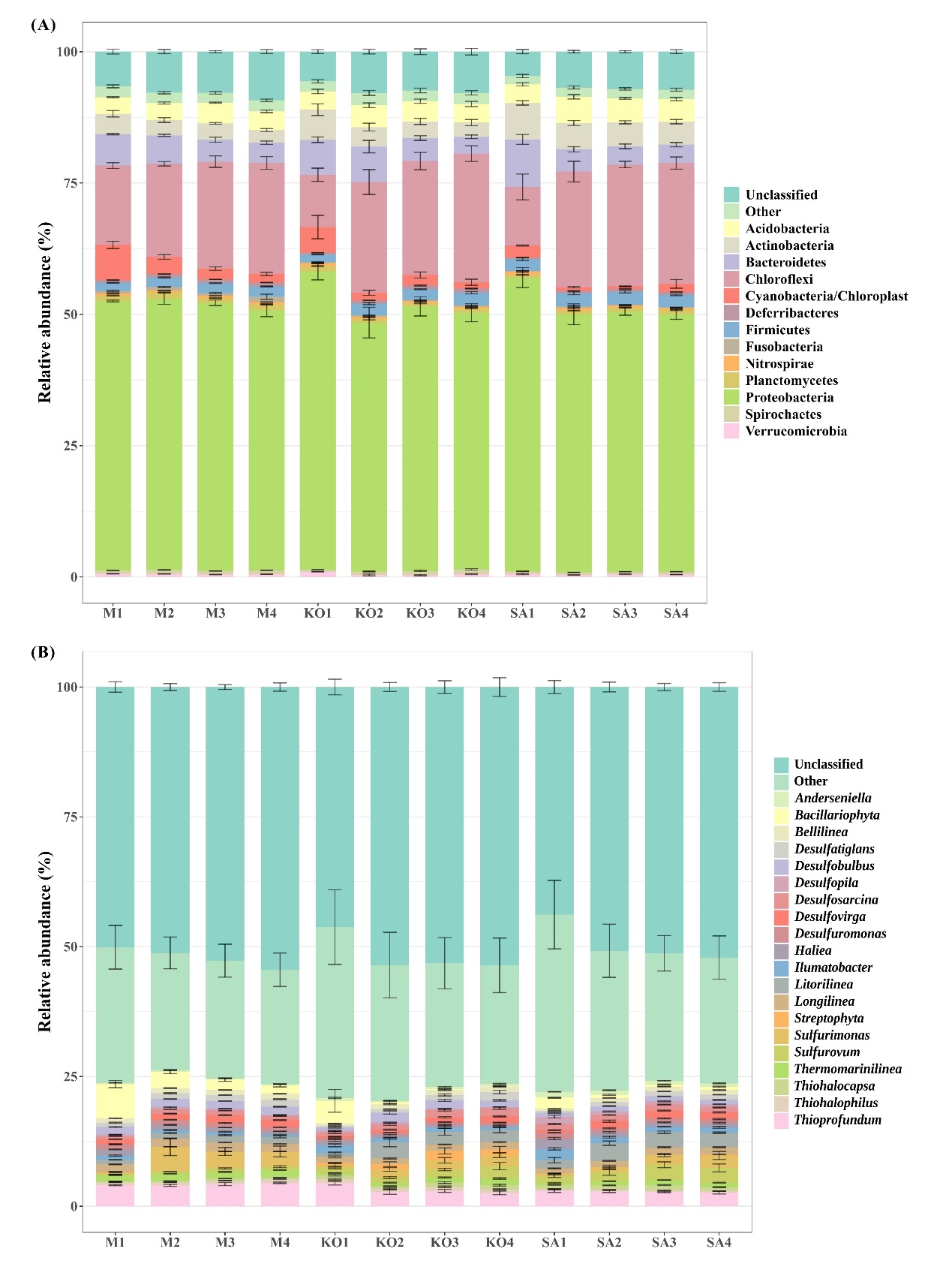


FIGURE S3 Taxonomic composition of bacterial communities in mangrove sediments at the phylum (A) and genus (B) levels. Different colors represent distinct phylum and genus. Sampling sites are denoted as M1, KO1, and SA1 (0–5 cm depth); M2, KO2, and SA2 (5–10 cm depth); M3, KO3, and SA3 (10–15 cm depth); and M4, KO4, and SA4 (15–20 cm depth)


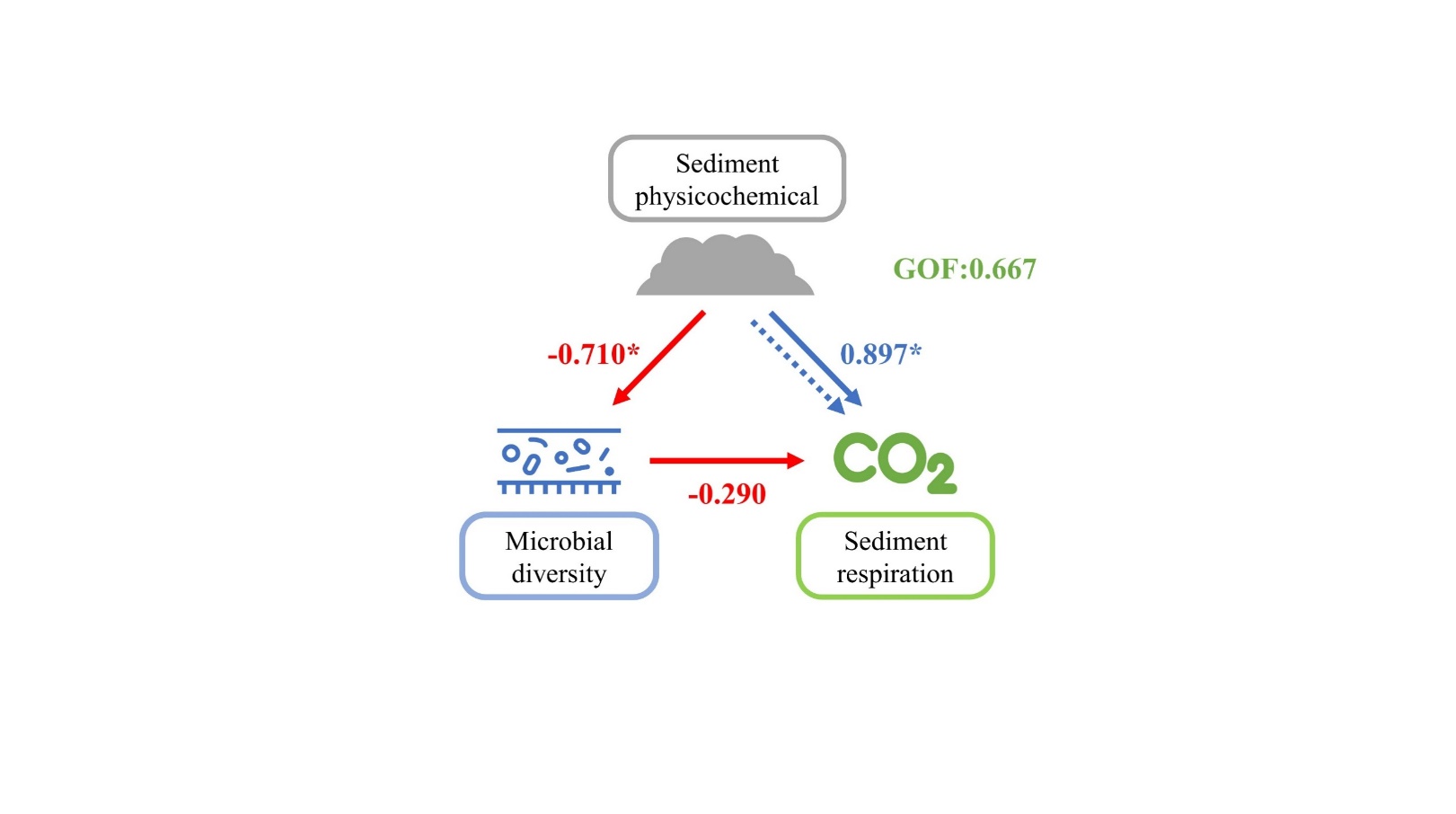


FIGURE S4 Partial least squares structural equation modeling (PLS-SEM) illustrating the relationships among sediment physicochemical properties, microbial community diversity, and sediment CO₂ flux in the studied mangrove habitats. Red and blue arrows represent positive and negative effects, respectively. Solid and dashed lines denote the direct and indirect effects, respectively. Numbers on the lines in the PLS-SEM model are the “total effect” values. The * indicate statistical significance at p < 0.05.
